# Pharmacy students’ motivations, satisfaction, and future career plans: A cross-sectional study exploring gender differences

**DOI:** 10.1371/journal.pone.0317896

**Published:** 2025-03-03

**Authors:** Nadine N. Abdelhadi, Ayat Al-Meanazel, Lidia Kamal Al-Halaseh, Mervat M. Alsous, Samah Al-Shatnawi, Anwar Abdel Qader Jaffal, Mohammad Yasin Mohammad, Rula M. Darwish

**Affiliations:** 1 Department of Clinical Pharmacy and Pharmacy Practice, Faculty of Pharmacy, Aqaba University of Technology, Aqaba, Jordan; 2 Department of Mathematics, Faculty of Science, Al Al-Bayt University, Al-Mafraq, Jordan; 3 Department of Pharmaceutical Chemistry, Faculty of Pharmacy, Mutah University, Al-Karak, Jordan; 4 Department of Clinical Pharmacy and Pharmacy Practice, Faculty of Pharmacy, Yarmouk University, Irbid, Jordan; 5 Department of Clinical Pharmacy, Faculty of Pharmacy, Jordan University of Science and Technology, Irbid, Jordan; 6 Department of Pharmacology and Medical Science, Faculty of Pharmacy, University of Petra, Amman, Jordan; 7 Pharmacological and Diagnostic Research Center, Faculty of Pharmacy, Al-Ahliyya Amman University, Amman, Jordan; 8 Department of Pharmaceutics and Pharmaceutical Technology, School of Pharmacy, The University of Jordan, Amman, Jordan; Lahore Medical and Dental College, PAKISTAN

## Abstract

**Background:**

The pharmacy profession has significantly changed over the years. Pharmacy students’ perceptions of their coursework and future career aspirations may vary in relation to gender.

**Objectives:**

The present study explored the motivations of pharmacy students to enter pharmacy school, their satisfaction with the academic program, future plans after graduation, and perceptions about the pharmacy profession in relation to gender.

**Methods:**

Data were collected using a cross-sectional descriptive validated questionnaire built by the research team. The study was conducted at twelve public and private universities offering pharmacy programs.

**Results:**

In total, 918 pharmacy students have completed the online questionnaire, with a 98% response rate. Most participants reported that family encouragement was a motive to enter pharmacy school. The results of the Chi-Squared Test indicated a significant difference between female and male participants with respect to the following motives: High school grades (p = 0.009), being good at science (p = 0.013), working with patients(p = 0.024), professional status (p = 0.014), working in a family business (p = 0.001) and job opportunities (p = 0.001). The majority of male participants and female participants perceived pharmacy jobs as prestigious jobs. In addition, male and female students believed that it was a profession with well-paid jobs.

**Conclusion:**

Females were significantly more motivated by their high school degrees, goodness at science, working with patients, and professional status to enter pharmacy school. Pharmacy students are satisfied enough with the academic program. Male and female pharmacists have different career aspirations in the pharmaceutical sectors. It is recommended that students be educated about career planning to help them accomplish their goals. Future research could benefit from longitudinal studies to explore changes in pharmacy students’ motivations, satisfaction, and career aspirations over time.

## Introduction

The pharmacy profession has significantly changed over the years, allowing it to effectively and securely meet society’s health needs [1–3]. The Pharmacy Curriculum Guidelines suggest competencies, such as leadership, decision-making, and communication skills, that pharmacists should develop to advance their knowledge and acquire critical clinical skills [4,5]. The Jordanian Accreditation and Quality Assurance Commission for Higher Education Institutions (AQACHEI) has a unified curriculum of pharmacy programs that would allow for the inclusion of such skills, as they encourage universities to include courses related to social and administrative pharmacy, marketing, and training in the pharmacy study plan. Moreover, activities that promote career awareness are essential for educating students about the program upon admission and about job options [6,7]. AQACHEI unified curriculum ensures that students acquire the program-level learning outcomes (PLOs), which are the knowledge, skills and attributes students are expected to attain by the end of a program of study [6,7].

Pharmacists’ duties were restricted to dispensing and compounding pharmaceutical preparations until the 20th century. The “seven-star chemist” concept was first presented by the World Health Organization (WHO) in 1997 [8]. It covered the various tasks that a pharmacist must perform, including those of caregiver, decision-maker, communicator, manager, teacher, lifelong learner, and leader [8].

Based on statistical data in Jordan, most pharmacy graduates are employed in the private sector, which includes privately owned community pharmacies, pharmaceutical industries, hospitals, medical supplies, and cosmetic companies. The remaining pharmacists are employed in the public sector [9].

Patient-centered pharmaceutical care services are not implemented in community pharmacy settings [10]. Hospital pharmacists represent a small percentage of the pharmacy workforce in Jordan [11]; unfortunately, most lack the clinical expertise and training required to operate in such an environment [12].

As for the pharmaceutical industry, it has experienced remarkable growth Since the 1980s. Recently, sixteen pharmaceutical companies account for almost 80% of pharmaceutical products in the market, which are exported to more than sixty international markets^11^, making the pharmaceutical sector a significant driver of Jordan’s economy [13,14]. The salary of pharmacists who work in pharmaceutical industries is higher than that of those who work in community pharmacies. However, job opportunities are much less than those available in other pharmaceutical sectors.

In the field of pharmacy, selecting a career domain is a challenging task. Several factors affect career choices, including job-related, personal, and family life factors [15,16]. Pharmacy students’ perceptions of their coursework and field of study may change as they continue their studies and acquire work experience. Nevertheless, pharmacies and educational institutions will be better equipped to prepare future pharmacists if they have a better understanding of students’ perceptions of the curriculum and presumptions about the field, given the crucial role that pharmacy plays in public health [17]. Therefore, educating students about career planning for the future could be beneficial in helping them accomplish their goals [2].

The main aim of the present study was to explore the motivations of Jordanian pharmacy students from twelve public and private universities to choose the pharmacy field, their satisfaction with the courses, future plans after graduation, and perceptions about the pharmacy profession in relation to gender.

## Materials and methods

### Ethical statement

The ethical approval was obtained from the Institutional Review Board (IRB) at Aqaba University of Technology on 12 November 2023 with the approval number AUT-23-012-03. Participants were provided written consent.

### Data collection

Data were collected using a cross-sectional descriptive questionnaire built and validated by the research team. The questionnaire consisted of 100 questions and included seven sections: Section 1: Demographic details, Section 2: Reasons behind choosing to study Pharmacy, Section 3: Satisfaction with the pharmacy course, Part 4: Students’ future plans following graduation, Part 5: Students’ perceptions about the pharmacy profession, Part 6: If dropping out was considered by students during their study and the reasons behind it, Part 7: Students training or working experience during their study. Answer options included yes or no answers, true or false answers and strongly agree, agree, neutral, disagree and strongly disagree answers.

The questionnaire was sent to four academics and four experienced pharmacists to ensure face validity. The questionnaire was modified according to their comments where appropriate before the main data collection started. To assess test-retest reliability, the questionnaire was administered on two different occasions to 20 randomly selected students. The second testing took place two weeks later. The data from pilot tests were not included in the final study survey analysis. The Test-retest reliability was calculated using Spearman’s correlation coefficient (r) and the rho-value was 0.81. Further statistical validation of the questionnaires reliability was also assured by calculating Cronbach’s Alpha value, which was found to be 0.78, which indicates acceptable test-retest reliability.

The study was conducted at twelve public and private universities offering pharmacy programs representing the north, central, and southern regions of Jordan. The students completed the online questionnaire in the presence of a member of the research team to answer any questions. The recruitment period started on 14-12-2023 and ended on 01-07-2024. Participants were provided written consent, and minors were excluded.

The study was carried out over a period of 6 months in 2024. The sample size was calculated with a 99% confidence level and a 4% margin of error based on the number of pharmacy students in Jordanian universities, according to the Jordan Pharmacists Association. A total number of 918 pharmacy students were included. Raosoft™ sample size calculator was used [18]. There were no exclusion criteria, as students were randomly recruited regardless of age, gender, and geographic location.

### Statistical analysis

The responses to all questions were encoded, entered, and analyzed using Jeffreys’s Amazing Statistics Program (JASP) Version 0.19.0 [19]. Responses were then presented as frequencies and percentages for categorical variables and as means and standard deviations for continuous variables. Comparisons between groups were performed using the Chi-Square Test, Independent Samples T-test, and One-way ANOVA. A *p*-value of < 0.05 was considered significant. The possible scores for each factor ranged from 5 to 1 (5 strongly agree to 1 strongly disagree). All hypothesis testing was two-sided.

## Results

### Characteristics of participants

In total, 918 pharmacy students completed the online questionnaire, with a 98% response rate. Most pharmacy students were females, 747 (81.4%), and the mean age of participants was 21.5 years (range 18–47) (Table 1).

**Table 1:** Demographic characteristics of participants (n = 918).

Age (years)	
Mean	21.5
Minimum	18
Maximum	47
Gender	N (%)
Male	171 (18.6)
Female	747 (81.4)
Type of School	
Public	580 (63.2)
Private	338 (36.8)
Classification of high school certificate	
National	881 (96)
IB	3 (0.3)
IGCSE	15 (1.6)
SAT	13 (1.5)
Turkey	1 (0.1)
UAE	1 (0.1)
Other	4 (0.4)
Country of high school certificate	
Inside Jordan	803 (87.5)
Outside Jordan	115 (12.5)
Academic year	
1st year	79 (8.6)
2nd year	91 (9.9)
3rd year	220 (24)
4th year	283 (30.8)
5th year	245 (26.7)
Currently working	
Yes	159 (17.3)
No	759 (82.7)
Training during study	
Yes	536 (58.4)
No	382 (41.6)

### Motivations for studying pharmacy in relation to gender

Most participants reported that family encouragement motivated them to enter pharmacy school. The results of the Chi-Squared Test indicated a significant difference between female and male participants with respect to the following motives: high school grades (p = 0.009), being good at science (p = 0.013), working with patients(p = 0.024), professional status (p = 0.014), family business (p = 0.001), and job opportunities (p = 0.001) (Table 2).

**Table 2:** Motivations for studying pharmacy in relation to gender.

	Male	Female	P value
	N (%)	N (%)	Chi-Squared Test
First university entrance choice	87 (51)	403 (54)	0.468
Friends encouragement	71 (41.5)	321 (43)	0.729
Family encouragement	148 (86.6)	680 (91)	0.075
Teacher advise	40 (23.4)	169 (22.6)	0.829
Influence of a visit to a university open day	23 (13.5)	105 (14.1)	0.837
Influence of a visit to a university	37 (21.6)	151 (20.2)	0.677
High school grades	107 (62.6)	543 (72.7)	0.009
Good at science	124 (72.5)	605 (81)	0.013
Working with patients	108 (63.2)	537 (71.9)	0.024
Closest profession to Medicine	112 (65.5)	488 (65.3)	0.967
Work in health-related field	148 (86.6)	670 (89.7)	0.234
Flexible working hours	114 (66.7)	503 (67.3)	0.866
Professional status	130 (76)	627 (84)	0.014
Family business	35 (20.5)	73 (9.8)	0.001
Job opportunities	105 (61.4)	358 (48)	0.001
Influence of a role model	59 (34.5)	213 (28.5)	0.122
Influence of media	46 (26.9)	218 (29.2)	0.552

### Satisfaction with the academic pharmacy program

Independent samples T-test was conducted. The possible scores for each factor ranged from 5 to 1 (5 Strongly Agree to 1 Strongly Disagree) (Table 3). The results of the Independent Samples T-Test showed a significant difference between female and male participants with respect to the following statements: Future salary plays a part in my satisfaction with studying pharmacy (p = 0.001) and The pharmacy study plan provides the necessary knowledge, skills, and competencies needed in the labor market (p = 0.026).

**Table 3:** Satisfaction with the academic pharmacy program.

Statement	Male	Female	Independent Samples T-Test
			P value
I clearly understood what pharmacy was when I entered the university.	4.006	3.888	0.181
I am satisfied with studying Pharmacy	3.778	3.704	0.457
I am satisfied with studying and progressing towards a career as a Pharmacist	3.848	3.795	0.571
Future salary plays a part in my satisfaction with studying Pharmacy	3.696	3.309	0.001
The number of credit hours in the pharmacy study plan is sufficient	3.749	3.640	0.236
The pharmacy study plan provides the necessary knowledge, skills, and competencies needed in the labor market	3.380	3.158	0.026
Pharmacy students should participate in extracurricular activities	3.865	3.977	0.152
I don’t regret entering pharmacy school	3.596	3.633	0.670

### Factors associated with satisfaction with the pharmacy academic program

Multiple linear regression was used to test which of the motivations for studying pharmacy significantly predicted the level of satisfaction among pharmacy students with the pharmacy academic program. The overall regression was statistically significant (R2 =  0.18, F(18, 812) =  9.715, p =  0.001). Table 4 shows which motivation is significantly associated with satisfaction with the pharmacy academic program. Also, it was found that future salary for pharmacy graduates significantly predicted overall satisfaction with the academic program (p <  0.000).

**Table 4:** Factors associated with satisfaction with the academic pharmacy program.

Factors	Coefficient (CO)	Regression model
		P value
High school grades	−0.262	0.002
Good at science	0.579	0.001
Job opportunities	0.318	0.001
Work in health-related field	0.478	0.001
Influence of a visit to a university open day	−0.319	0.018
Family business	−0.255	0.048
First choice	0.172	0.036
Professional status	0.239	0.045

It was found that the level of satisfaction with the academic program would increase by the following factors: being good at science (CO =  0.579), working in a health-related field (CO =  0.478), having job opportunities (CO =  0.318), having professional status (CO =  0.239), and having pharmacy as a first choice (CO =  0.172).

The level of satisfaction with the academic program would decrease due to the following factors: family business (CO =  −0.255), high school grades (CO =  −0.262), and the influence of a visit to a university open day (CO =  −0.319).

In conclusion, good knowledge of science increases the level of satisfaction in the academic program by 0.579. Meanwhile, having a family business decreases the level of satisfaction in the academic program by 0.255.

### Satisfaction with the pharmacy program by academic year

One-way ANOVA showed a significant difference among academic years in relation to satisfaction with pharmacy academic programs, F (4,913) = 5.575, P value = 0.001. Post hoc testing revealed a significant difference between fourth-year students (Mean = 3.466, SD = 1.238) having less satisfaction than first-year (Mean = 3.848, SD = 1.00), second-year (Mean = 3.967, SD = 0.983), third-year (Mean = 3.864, SD = 1.139), and fifth-year (Mean = 3.743, SD = 1.171) students.

Students were asked if they had any suggestions to improve their educational experience. Two hundred (22%) students provided suggestions. Most of them (152; 76%) suggested increasing the number of practical courses during their study and increasing the number of field visits (114; 57%), respectively.

### Dropping out the course by academic year

Students were asked if they considered dropping out of the course and in which year. The majority of those who considered dropping out (491; 53.5%) were in the 2nd year (148; 30%), followed by the 1st year (141; 29%), 3rd year (139; 28%), 4th year (48; 10%) and 5th year (15; 3%). The major reasons for dropping out were mistaken choice (140; 29%), followed by academic difficulties (132; 27%), personal problems (120; 24%), and financial problems (99; 20%).

### Future career aspirations in relation to gender

The Chi-Square Test was utilized to study future career aspirations in relation to gender (Table 5). Answer options included yes or no. Results showed significant differences between females and males in their future career aspirations regarding some aspects, including hospital dispensing pharmacists, medical promotion, working in a family business, owning a pharmacy, and working outside their home country.

**Table 5:** Future career aspiration in relation to gender.

Future career aspiration	Male	Female	Chi-Square Test
	N (%)	N (%)	P value
Hospital dispensing pharmacist	87 (50.9)	465 (62.2)	0.006
Hospital clinical pharmacist	89 (52)	430 (57.6)	0.189
Community pharmacy	88 (51.5)	380 (50.9)	0.889
Medical Promotion	96 (56.1)	318 (42.6)	0.001
Research	108 (63.2)	396 (53)	0.016
Academia	89 (52)	358 (48)	0.331
Family business	41 (24)	98 (13.1)	0.001
Regulatory affairs and drug registration	72 (42.1)	297 (39.8)	0.572
Own a Pharmacy	133 (77.8)	513 (68.7)	0.019
Pharmaceutical industry	110 (64.3)	494 (66.1)	0.654
Inside home country	98 (57.3)	490 (65.6)	0.042
Outside home country	110 (64.3)	386 (51.7)	0.003
Not sure where I want to work	85 (49.7)	393 (52.6)	0.493
Do not want to work	10 (5.9)	78 (10.4)	0.066

### Perceptions about the pharmacy profession in relation to gender

The majority of male participants (127, 74.3%) and female participants (536, 71.8%) perceived pharmacy jobs as prestigious jobs. In addition, male students (76, 44.4%) and female students (310, 41.5%) believed that it is a profession with well-paid jobs. Few male participants (13, 7.6%) and female participants (67, 9%)) think that pharmacists should only work in hospitals, and only 7% and 7.8% of female and male students, respectively, believed that pharmacists should only work in community pharmacies. The results of the Chi-Squared Test did not indicate a significant difference between female and male participants

### Expected level of monthly salary in relation to gender

The majority of pharmacy students expect a monthly salary in the range of 705–988 USD.

#### Male students.

Most male pharmacy students (55, 32%) expect a monthly salary range from 705–988 USD. Twenty per cent (34) of males expect a monthly salary of more than 1411 USD, and 18% and 20% of participants expect a monthly salary range from 423–705 USD and 988–1411 USD.

#### Female students.

Most female students (235, 31%) expect a monthly salary between 705 and 988 USD. Only 9% of female pharmacy students expect a monthly salary of more than 1411 USD, and 28% and 17% of participants expect a monthly salary between 423 and 705 USD and 988–1411 USD.

## Discussion

The increasing number of pharmacy schools initiated a competition to attract more students to their pharmaceutical programs. The drop in the total applications made it inevitable to identify the influencing motivating factors for studying pharmacy. The revealed factors include the satisfaction level with the curriculum and the program learning objectives, the expectations of the profession after graduation, and the feedback they gave to high-school graduates. The main objective of the current study is to analyze these motivating factors to meet the students’ ambitions and improve the teaching output.

Regarding the study results, higher responses from female pharmacy students are expected since this reflects the gender distribution of pharmacy students in Jordan [20]. The age means matched the Jordanian education system, where qualified school students could join university faculties after successfully acquiring the high secondary certificate, where they aged 17–18 years. The responses of older students (reaching 47 years) are explained by the recognition of prior education. For example, individuals who obtained a pharmacy diploma are allowed to equivalence the diploma courses with matched course learning objectives (CLO) and proceed to finish the requirements of the bachelor’s degree. This policy was validated by the Ministry of Jordanian Higher Education but discontinued in September 2022.

Getting more responses from students in their 3^rd^ –5^th^ academic year increases the value of the study output because they could give deep insight into the curriculum, teaching system, and the internal and external motivating factors to continue in the program [21,22]. According to the pharmacy curriculum, the training courses (equivalent to 6 credit hours) require the students to pass at least 90 out of an average of 165 credit hours. Thus, it is expected to have a high percentage of students enrolled in field-training programs.

The questioned motivating factors were analyzed according to the student’s gender. A comparable percentage of the respondents have declared themselves motivated by the interrogated factors. However, females were significantly more encouraged by their high school degrees, goodness at science, working with patients, and professional status. The decision made by male students to register in pharmacy programs was significantly affected by their family business and job opportunities. Few published studies have analyzed the gender differences in choosing pharmacy as a major. A Malaysian study concluded that females were influenced mainly by work conditions and professional attributes [23].

From another perspective of the analyzed data, the most influential factor was family encouragement, which is considered an external motivator, followed by working in health-related fields and professional status factors. The viewpoints of Jordanian students mirrored those from other nationalities, where the pharmacy profession’s reputation was one of the top career aspirations [24]. Furthermore, the role of pharmacists has expanded significantly to be more involved in the healthcare system, particularly after the COVID-19 pandemic. Pharmacists stood at the forefront to overcome the global challenges, which deepened the crucial contribution to the health system [25].

Career opportunities in pharmacy and the availability of multiple work fields have opened multiple job choices to skilled and well-educated graduates. Male and female pharmacists have different preferences in the pharmaceutical sectors. This might refer to their different responses to the various facets of their jobs, overall job satisfaction, different responses to stimuli, work-related incentives, and others [26–33].

It is encouraging to find that enrolled students are satisfied enough with the academic program provided by the pharmacy faculties in Jordanian academic institutions. They declare having a good perception of pharmacy PLOs, knowledge, skills, competencies, and career pathways. The overall satisfaction of pharmacy students has been a thorny topic. A study that took place in 2020 at the University of Kentucky revealed varying levels of disengagement, burnout, and emotional exhaustion between students in different professional years [26]. An earlier study performed in a Malaysian university showed dissatisfied students regarding some elements of the curriculum, such as overlapping in the practical courses and the inclusion of the Malaysian language [27].

Regarding pharmacy students’ perceptions of the pharmacy profession, most of the participants, regardless of their gender, recognized pharmacy as a prestigious and well-paid job. The results matched an Australian study that analyzed pharmacists’ job preferences. Almost half of the Australian participants (51.33%) were employed in the community pharmacy sector, and 25.22% had jobs in hospital pharmacies. The pharmaceutical industry and primary care units were occupied with the lowest percentage [28].

According to the income expectations and the monthly salary influence on choosing the pharmacy profession, the obtained results were parallel with previously published reports that confirm that salary and benefits are important factors in career selection [29].

Nationally, before the implementation of national and international academic accreditations, there was a need to improve pharmacy education to meet the student’s expectations and labour market need [30]. To the authors’ best knowledge, this is the first study to measure students’ satisfaction with the academic program in almost all the Jordanian public and private colleges of pharmacy after fulfilling the accreditation requirements.

### Limitations

Limitations are due to the cross-sectional design and include the selection bias and the reliance on self-reporting of data. Another limiting factor is the low response rate among students with non-Jordanian high school certificates, who are not well-presented in the current study. Moreover, a small percentage (12%) of the participants acquired their high school certificate from outside Jordan. Thus, the motivating factors for this group were not fully addressed. Further analyses are encouraged to figure out the effects of other possibly incorporating factors such as economic status and cultural background.

## Conclusion

Females were significantly more encouraged by their high school degrees, goodness at science, working with patients, and professional status. The decision made by male students to register in pharmacy programs was significantly affected by their family business and job opportunities. Male and female pharmacists have different career aspirations in the pharmaceutical sectors. Enrolled students are satisfied enough with the academic program provided by the pharmacy faculties in Jordanian academic institutions. They declare having a good perception of pharmacy program learning outcomes, knowledge, skills, competencies, and career pathways. It is recommended that students be educated about career planning to help them accomplish their goals. Future research could benefit from longitudinal studies to explore changes in pharmacy students’ motivations, satisfaction, and career aspirations over time.

## References

[1] Crass RL, Romanelli F. Curricular reform in pharmacy education through the lens of the flexner report of 1910. Am J Pharm Educ. 2018;82(7):6804. doi: 10.5688/ajpe6804 30323394 PMC6181160

[2] Arbab AH, Eltahir YAM, Elsadig FS, Yousef BA. Career preference and factors influencing career choice among undergraduate pharmacy students at University of Khartoum, Sudan. Pharmacy (Basel). 2022;10(1):26. doi: 10.3390/pharmacy10010026 35202075 PMC8880343

[3] Forsyth P, Radley A, Rushworth GF, Marra F, Roberts S, O’Hare R, et al. The collaborative care model: realizing healthcare values and increasing responsiveness in the pharmacy workforce. Res Social Adm Pharm. 2023;19(1):110–22. doi: 10.1016/j.sapharm.2022.08.016 36100521

[4] Alefan Q, Alsmadi MM. Pharmacy education in Jordan: updates. Int J Pharm Pract. 2017;25(6):418–20. doi: 10.1111/ijpp.12344 28211595

[5] Saseen JJ, Ripley TL, Bondi D, Burke JM, Cohen LJ, McBane S, et al. ACCP clinical pharmacist competencies. Pharmacotherapy. 2017;37(5):630–6. doi: 10.1002/phar.1923 28464300

[6] Abdelhadi NN, Wazaify M, Darwish Elhajji FW, Basheti IA. Doctor of pharmacy in Jordan: students’ career choices, perceptions and expectations. J Pharm Nutr Sci. 2014;4(3):213–9. doi: 10.6000/1927-5951.2014.04.03.7

[7] McMullen J, Arakawa N, Anderson C, Pattison L, McGrath S. A systematic review of contemporary competency-based education and training for pharmacy practitioners and students. Res Social Adm Pharm. 2023;19(2):192–217. doi: 10.1016/j.sapharm.2022.09.013 36272964

[8] Developing pharmacy practice: A focus on patient care: Handbook. World Health Organization. 2006 [cited 2024 Aug 9]. Available from: https://apps.who.int/iris/handle/10665/69399

[9] The Hashemite Kingdom of Jordan the Higher Health Council the National Strategy for Health Sector in Jordan, 2015. [cited 2024 Aug 9]. Available from: https://extranet.who.int/countryplanningcycles/sites/default/files/planning_cycle_repository/jordan/jordan_national_health_sector_strategy_2015-2019_.pdf.HigherHealthCouncil

[10] Nazer LH, Tuffaha H. Health care and pharmacy practice in Jordan. Can J Hosp Pharm. 2017;70(2):150–5. doi: 10.4212/cjhp.v70i2.1649 28487583 PMC5407425

[11] Al-Wazaify M, Matowe L, Albsoul-Younes A, Al-Omran OA. Pharmacy education in Jordan, Saudi Arabia, and Kuwait. Am J Pharm Educ. 2006;70(1):18. doi: 10.5688/aj700118 17136159 PMC1636892

[12] Bader LR, McGrath S, Rouse MJ, Anderson C. A conceptual framework toward identifying and analyzing challenges to the advancement of pharmacy. Res Social Adm Pharm. 2017;13(2):321–31. doi: 10.1016/j.sapharm.2016.03.001 27117185

[13] Alomari MW, Alomari M, Saqfalhait N. Analyzing the structure of Jordanian pharmaceutical industry. Eur J Soc Sci. 2015;49(1).

[14] Rollins BL, Perri M. Pharmaceutical marketing. Jones & Bartlett Learning; 2014.

[15] Al-Qudah R, Abuhussein R, E H, Rezeq M, Basheti I. Factors influencing career choice among undergraduate pharmacy students at a private university in Jordan. Pharm Educ. 2019;19(1):56–61.

[16] Khurshid F, Alharbi F, Almohydib A, Malik S, Al-Omar H, Alsultan M, et al. Preparatory year students’ perception of pharmacy profession as a career choice: a cross-sectional study. Braz J Pharm Sci. 2023;59:e21476.

[17] Keshishian F, Brenton BP. Pharmacy students’ perceptions of their curriculum and profession: implications for Pharmacy Education. Pharm Educ. 2011;11.

[18] Raosoft I. Sample Size Calculator by Raosoft Inc. [Online] [cited 2023 May 15]. Available from: http://www.raosoft.com/samplesize.html

[19] JASP Team. JASP (Version 0.19.0) [Computer software]. 2024.

[20] Shudifat RM, Mosleh S, Almakhzomi S, Al Shdifat M, Alnajar M, Alkhawaldeh JM, et al. Measuring the knowledge and perception of Jordanian health science students towards self-prescribed medications: a descriptive analysis study. J Pharm Health Serv Res. 2023;15(1). doi: 10.1093/jphsr/rmad049

[21] Perraudin C, Brion F, Bourdon O, Pelletier-Fleury N. The future of pharmaceutical care in France: a survey of final-year pharmacy students’ opinions. BMC Clin Pharmacol. 2011;11:6. doi: 10.1186/1472-6904-11-6 21612642 PMC3115856

[22] Shen G, Fois R, Nissen L, Saini B. Course experiences, satisfaction and career intent of final year pre-registration Australian pharmacy students. Pharm Pract (Granada). 2014;12(2):392. doi: 10.4321/s1886-36552014000200004 25035715 PMC4100949

[23] Loo JSE, Lim SW, Ng YK, Tiong JJL. Pharmacy students in private institutions of higher education: motivating factors when studying pharmacy and influences on university choice. Int J Pharm Pract. 2017;25(6):429–37. doi: 10.1111/ijpp.12352 28211115

[24] Hammoudi Halat D, Al-Jumaili AA, Ahmed KK, Rahal M, Hamad A, Darwish RM, et al. Academic entitlement among pharmacy students in the Arab World: a multi-national exploratory study. Am J Pharm Educ. 2024;88(2):100640. doi: 10.1016/j.ajpe.2023.100640 38181969

[25] Hartis C. “Pharmacy is a Profession Devoted to Helping Patients”: Investigating the Ever-Transforming Role of Pharmacists Post COVID-19 (Master’s thesis, University of Cincinnati).

[26] Fuller M, Schadler A, Cain J. An investigation of prevalence and predictors of disengagement and exhaustion in Pharmacy Students. Am J Pharm Educ. 2020;84(10):ajpe7945. doi: 10.5688/ajpe7945 33149329 PMC7596598

[27] Al-Efan A, Mohamed MHN, Awaisu A, Tariq AR, Ab Rahman J. Students’ perspectives on pharmacy curriculum in a Malaysian University. Published online 2009 Jan 1.

[28] Thai T, Lancsar E, Spinks J, Freeman C, Chen G. Understanding Australian pharmacy degree holders’ job preferences through the lens of motivation-hygiene theory. Soc Sci Med. 2024;348:116832. 38569288 10.1016/j.socscimed.2024.116832

[29] Hussain M, Sahudin S, Fauzi SM, Manaf NA, Wahab MSA. Exploring pharmacy students chosen career path: a year-on-year perspective. High Educ. 2020;81(6):1257–72. doi: 10.1007/s10734-020-00610-6

[30] M Wazaify, A Albsoul-Younes, N Hakooz. Improving pharmacy education at the Public Faculties of Pharmacy in Jordan: students’ experience and expectations. Pharm Educ. 2010;10.

[31] Carvajal MJ, Armayor GM, Deziel L. The gender earnings gap among pharmacists. Res Social Adm Pharm. 2012;8(4):285–97. doi: 10.1016/j.sapharm.2011.06.003 21958465

[32] Carvajal MJ, Popovici I, Hardigan PC. Gender differences in the measurement of pharmacists’ job satisfaction. Hum Resour Health. 2018;16(1):33. doi: 10.1186/s12960-018-0297-5 30064513 PMC6069841

[33] Ashal N, Masa’deh R, Twaissi NM. The impact of learning organization on intrapreneurship: the Case of Jordanian Pharmaceutics. Sustainability. 2023;15(16):12211. doi: 10.3390/su151612211

